# Research on the Dielectric Constant Sensor System Based on the Short-Ended Transmission Line

**DOI:** 10.3390/s26154830

**Published:** 2026-07-30

**Authors:** Haoyang Shi, Xuchun Zhang, Chuan Sheng, Lin Huang, Kun Wang, Xikang Li

**Affiliations:** Air Defense and Anti-Missile Academy, Air Force Engineering University, Xi’an 710051, China; zxcxueshu@126.com (X.Z.); linhuang125@163.com (L.H.); 17765073967@163.com (K.W.); lixikang314@163.com (X.L.)

**Keywords:** dielectric constant, sensor system, short-ended transmission line, resonance frequency

## Abstract

This paper presents a sensor system for measuring the dielectric constant of materials, with a control circuit, a broadband coupler and a sensing probe as its main components. The entire system can independently measure the dielectric constant of powdered solid materials and liquid materials without relying on any auxiliary equipment. According to the transmission line design theory, the sensing probe utilizes a short-ended transmission line based on air coplanar waveguide. By introducing a parallel resistor at the input of the transmission line, material under test (MUT) with different dielectric constant can directly influence the resonance frequency of the transmission line’s reflection coefficient. The dielectric constant can be inverted through the measurement of the resonance frequency. This paper systematically analyzes the sufficient condition for the validity of the conclusion and discusses interference factors that may affect the measurement result, such as variation in resistor value and difference in material loss. Subsequently, a dedicated control circuit was designed and manufactured by integrating a control chip, an RF chip, and a detector, thus forming a low-cost, portable sensor system together with the sensing probe and coupler. Experimental tests were conducted on six different MUTs, and the results confirmed that the system has the capability to accurately characterize the dielectric constant of powdered solid and liquid material. Compared to other sensor systems, the proposed system features a simplified design and higher measurement precision, indicating its broad applicability in radio frequency identification.

## 1. Introduction

The dielectric constant, as a core electromagnetic parameter of a material, is a key indicator for measuring its molecular polarization capacity and interaction with electric fields [1,2]. Currently, microwave-based methods for determining the dielectric constant of materials are extensively categorized in the literature into resonant techniques such as cavity perturbation and dielectric resonators, and broadband transmission and reflection techniques such as coaxial line, waveguide, and free-space methods [3,4]. Beyond this primary classification, comprehensive taxonomies also account for material states including solids, liquids, metals, and lossy media, as well as advanced structures such as negative-index materials. In the context of resonant approaches, sensitivity is typically high but often relies on indirect fitting of frequency shifts, whereas transmission and reflection methods provide broadband capability yet usually require vector network analyzers for complex scattering parameter extraction. The resonant method determines the dielectric constant of materials by measuring the resonant frequency shift or resonance depth variation after MUT loading [5,6,7,8].

Recent progress in dielectric constant sensing for low-loss materials has attracted considerable attention due to their critical roles in integrated circuits and microwave devices, with representative works reporting novel strategies based on cylindrical resonant cavities and refined perturbation corrections to enhance measurement accuracy and accommodate small-sized samples [9]. Currently, research on microwave sensor systems has adopted various strategies, including the application of metamaterial technology to load split-ring resonators (SRRs) or complementary split-ring resonators (CSRRs) on microstrip lines for the precise determination of the dielectric constant of composite materials [10,11,12]. To enhance system sensitivity, shunt-connected series LC resonators have been loaded onto transmission lines [5], and AI-assisted detection methods have been employed [13]. To address the practical issue that microwave sensor systems rely on auxiliary equipment for measurement, a novel low-cost microwave sensor system based on a software-defined radio platform has been proposed in a recent report, which eliminates the need for advanced measurement devices such as a vector network analyzer (VNA) for system characterization [6]. Notably, stand-alone and portable instruments have also been developed to perform reliable dielectric constant measurements without specialized laboratory setups, serving as exemplary implementations of independent dielectric measurement systems [14]. Owing to their advantages of low cost and high measurement precision, microwave sensor systems can be applied not only to the determination of the dielectric constant of materials but also to non-invasive blood glucose level monitoring [15], temperature detection [16], and water quality assessment [17]. Conventional microwave sensor systems primarily adopt an indirect measurement method. This method involves fitting the relationship between variations in material characteristics and resonant frequency or phase through potential coupling mechanisms. A scalar method for accurately calculating the reactive component values in the microwave and millimeter-wave frequency bands has been presented in previous studies and validated via Smith chart simulations [18]. Additionally, a method combining short-ended and open-ended transmission lines for measuring the dielectric constant of liquids has been proposed in [19]. However, this method fails to accurately extract the dielectric constant information of the MUT due to the fringing field effects of open-ended transmission lines.

Building on the aforementioned research, this paper presents a sensor system capable of measuring the dielectric constant of both liquid and powdered solid materials. The advantages of this sensor system lie in its measurement principle, which enables the direct calculation of the dielectric constant from measurement data via a physically transparent analytical formula. This analytical expression, derived explicitly for the reflection resonator configuration, complements the well-established resonant sensing framework by providing a direct relationship between the permittivity and the measurable resonance period. The system also offers fully independent measurement operational capability.

The specific structure of this paper is outlined as follows. Section 2 focuses on the working principle of the sensing probe for the direct measurement of dielectric constant and analyzes the relationship among the system error of the sensor system, the MUT and the operating frequency. Section 3 addresses potential non-ideal factors that may arise during actual fabrication. Section 4 elaborates on the working principle of the entire sensor system, provides experimental validation and conducts a comparative analysis with existing research. Finally, Section 5 summarizes the main work and conclusions of this study. It should be noted that the core innovation of this paper lies in the proposed direct scalarization measurement method. Thus, this paper centers on the principle and implementation of this method, specifically how to realize the direct measurement of dielectric constant by using a short-ended transmission line with a parallel resistor. For this reason, the description of the backend hardware circuit system is appropriately simplified.

## 2. Analysis of Measurement Principle

**Figure 1:**
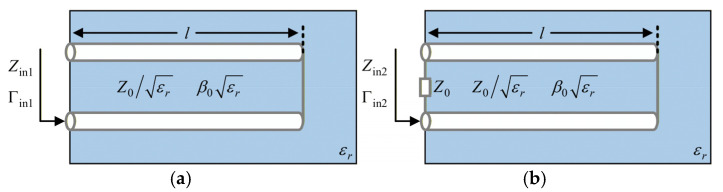
A terminal-short-ended transmission line fully immersed in an environment with a dielectric constant of $\varepsilon_{r}$. (**a**) Input end without parallel resistance; (**b**) Input end with a parallel resistance of nominal value $Z_{0}$.

As shown in Figure 1a, consider a transmission line of length l, which has a characteristic impedance of $Z_{0}$ and a phase constant of $\beta_{0}$ in vacuum. If this transmission line is short-ended at its terminal and completely placed in an environment with a dielectric constant of $\varepsilon_{r}$, the characteristic parameters of the short-ended transmission line will be directly affected by $\varepsilon_{r}$, leading to changes in the characteristic impedance and phase constant, which should be adjusted to $Z_{0}/\sqrt{\varepsilon_{r}}$ and $\beta_{0}\sqrt{\varepsilon_{r}}$, respectively. Based on microwave transmission line theory, the input impedance $Z_{\mathrm{in}1}$ of the short-ended transmission line is given by:

(1) $$Z_{\mathrm{in}1}=j\frac{Z_{0}}{\sqrt{\varepsilon_{r}}}\tan\left(\beta_{0}l\sqrt{\varepsilon_{r}}\right)$$

Taking the reciprocal of (1) yields an input admittance $Y_{\mathrm{in}1}$ of the short-ended transmission line as:

(2) $$\begin{array}{l} Y_{\mathrm{in}1}=-j\frac{\sqrt{\varepsilon_{r}}}{Z_{0}}\cot\left(\beta_{0}l\sqrt{\varepsilon_{r}}\right)\\ \;=-jb_{s}Y_{0} \end{array}$$

In (2), $Y_{0}=1/Z_{0}$ represents the characteristic admittance of the transmission line in vacuum, and $b_{s}=\sqrt{\varepsilon_{r}}\cot\left(\beta_{0}l\sqrt{\varepsilon_{r}}\right)$ denotes the input susceptance of the short-ended transmission line. It can be observed that $b_{s}$ contains information on the dielectric constant, and thus measuring $b_{s}$ is expected to facilitate the determination of the dielectric constant. The reflection coefficient $\Gamma_{\mathrm{in}1}$ at the input of the short-ended transmission line is given by:

(3) $$\Gamma_{\mathrm{in}1}=\frac{Y_{0}-Y_{\mathrm{in}1}}{Y_{0}+Y_{\mathrm{in}1}}=\frac{1+jb_{s}}{1-jb_{s}}$$

From (3), it is evident that since $b_{s}$ is a real number, the magnitude of the reflection coefficient of the short-ended transmission line is always 1. Therefore, it does not reflect the dielectric constant of the material. In this case, $\varepsilon_{r}$ can only be characterized by the phase of the reverse reflection coefficient. Since phase measurement falls under the category of vector measurement, vector measurement is often associated with higher costs. This paper proposes a scalar measurement method that makes the dielectric information accessible without vector-based phase detection. As shown in Figure 1b, a resistor with a nominal value of $R=Z_{0}$ is connected in parallel at the input of the short-ended transmission line, so that the input admittance $Y_{\mathrm{in}2}$ become:

(4) $$Y_{\mathrm{in}2}=Y_{\mathrm{in}1}+Y_{0}$$

The reflection coefficient $\Gamma_{\mathrm{in}2}$ at the input of the short-ended transmission line after adding the parallel resistor is given by:

(5) $$\Gamma_{\mathrm{in}2}=\frac{Y_{0}-\left(Y_{\mathrm{in}1}+Y_{0}\right)}{Y_{0}+\left(Y_{\mathrm{in}1}+Y_{0}\right)}=\frac{jb_{s}}{2-jb_{s}}$$

From (5), it is evident that the magnitude of the reflection coefficient for the short-ended transmission line with a parallel resistor is no longer constant, but is directly related to the dielectric constant, thus laying the foundation for the scalar measurement of dielectric constant. By combining (2), the modulus of the reflection coefficient in (5) can yield:

(6) $$\left|\Gamma_{\mathrm{in}2}\right|=\frac{\left|\sqrt{\varepsilon_{r}}\cot\left(\beta_{0}l\sqrt{\varepsilon_{r}}\right)\right|}{\left|2-j\sqrt{\varepsilon_{r}}\cot\left(\beta_{0}l\sqrt{\varepsilon_{r}}\right)\right|}$$

The numerator in (6) is a real number containing the cotangent function, while the denominator is always a positive real number. It can thus be inferred that the magnitude of the reflection coefficient exhibits periodic zeros, which correspond to the resonant points. Setting the numerator to zero yields the following equation:

(7) $$\beta_{0}l\sqrt{\varepsilon_{r}}=\frac{\pi }{2}+k\pi$$

where k=0, 1, 2 ⋯, and the phase constant is defined as $\beta_{0}=2\pi f/c$. Given that the frequency difference between two adjacent resonance points is Δf, substitution into (7) yields:

(8) $$\frac{2\pi \Delta f}{c}\;l\sqrt{\varepsilon_{r}}=\frac{\pi }{2}$$

Further rearranging (8) yields the direct analytical expression as follows:

(9) $$\varepsilon_{r}=\frac{c^{2}}{4l^{2}\Delta f^{2}}$$

Equation (9) indicates that, with a shunt resistor connected at the input of the short-ended transmission line, the dielectric constant of the MUT can be directly determined by measuring the frequency spacing between adjacent resonant nulls of the reflection coefficient. The proposed method thus has no inherent theoretical upper limit for the measurable dielectric constant within the constraints of the hardware frequency resolution and bandwidth.

From a physical perspective, the proposed structure can be interpreted as a reflection resonator. The shunt resistor connected at the input of the short-ended transmission line provides a matched coupling between the line and the external measurement circuit, whereas the short-ended line section itself acts as the frequency-selective element. At frequencies where the electrical length of the short-ended line is an integer multiple of a half-wavelength, the line presents a high input impedance and the total reflection coefficient approaches zero, giving rise to the resonant nulls observed in the magnitude spectrum. The frequency spacing between adjacent nulls, denoted as Δf, corresponds to the free spectral range of this reflection resonator and is directly related to the dielectric constant of the surrounding material via the analytical expression in (9). This resonant interpretation clarifies the underlying physical mechanism and connects the proposed method to the well-established framework of reflection-type resonant sensors, while the explicit analytical formula derived herein represents the distinctive contribution of this work.

**Figure 2:**
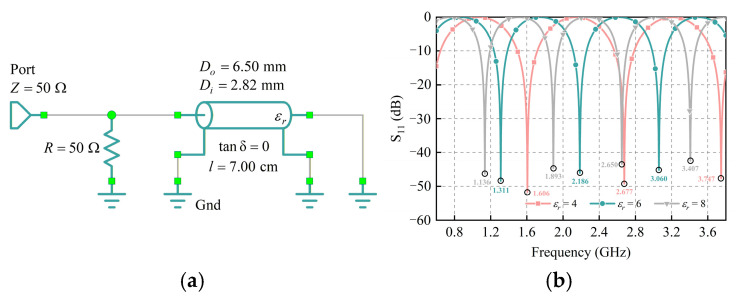
Ideal circuit model and simulation results. (**a**) Short-ended transmission line circuit model constructed using a coaxial line with a parallel resistor; (**b**) Simulation results of the reflection coefficient magnitude for different dielectric constant.

To validate the feasibility of this scalar measurement method, simulation verification was carried out for the ideal circuit using AWR Design Environment 17simulation software, with the corresponding circuit model depicted in Figure 2a. A coaxial line was adopted as the short-ended transmission line to facilitate the adjustment of the dielectric constant of the intermediate dielectric layer, thereby simulating the transmission line in environments with different materials. When the dielectric constant of the dielectric layer is $\varepsilon_{r}=1$, the outer conductor diameter is $D_{o}=6.50\;\mathrm{mm}$, and the inner conductor diameter is $D_{i}=2.82\;\mathrm{mm}$, the characteristic impedance of the coaxial line can meet the requirement of 50 Ω. From (9), it is clear that for a given dielectric constant, the length of the transmission line l is inversely proportional to the frequency difference Δf between adjacent resonance frequencies. That is, the shorter the transmission line, the greater the frequency difference between adjacent resonance frequencies. During circuit simulation, the length of the coaxial line was set to l=70.00 mm, the simulation frequency band was set to 0.6 GHz–3.8 GHz, and the step size was 0.001 GHz. Throughout the simulation, the loss tangent of the dielectric layer was set to tanδ=0. By adjusting $\varepsilon_{r}$ to different values, the variation in the magnitude of the input reflection coefficient was observed, with results shown in Figure 2b. The simulation results indicate that when $\varepsilon_{r}$ varies, the magnitude of the reflection coefficient exhibits resonances at different frequencies. When $\varepsilon_{r}$ is set to 4, 6, and 8, the frequency differences between adjacent resonance frequency are 1.071 GHz, 0.875 GHz, and 0.757 GHz, respectively. The calculated dielectric constant obtained by substituting into (9) are 4.003, 5.998, and 8.013, respectively. The calculated values are nearly equal to the set values, demonstrating the effectiveness of the scalar measurement method proposed in this paper.

**Figure 3:**
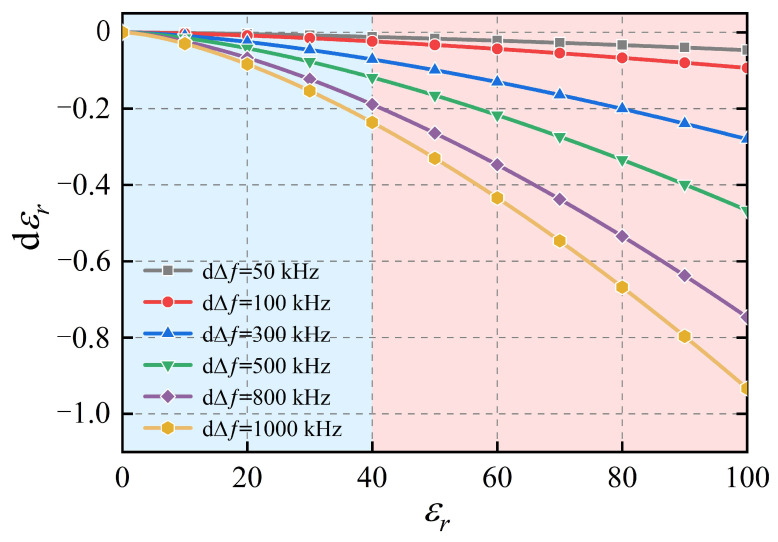
Systematic error of the scalar method under various frequency measurement errors.

**Figure 4:**
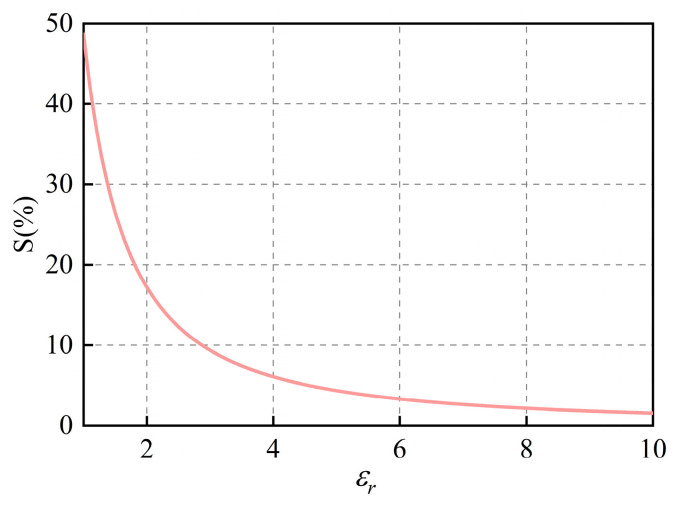
The sensitivity variations with respect to different dielectric constant.

To further analyze the systematic error of the proposed scalar measurement method, differentiating both sides of Equation (9) yields the following:

(10) $$d\varepsilon_{r}=-\frac{4l{\left(\sqrt{\varepsilon_{r}}\right)}^{3}}{c}d\Delta f$$

where dΔf represents the measurement error of the frequency detection device. From (10), it can be seen that the systematic error of the scalar method is also related to the dielectric constant. When dΔf is fixed, the larger the dielectric constant of the MUT, the greater the resultant systematic error. When the length of the short-ended transmission line is 70.00 mm, the relationship between the systematic error of the scalar method and the dielectric constant is shown in Figure 3. As the dielectric constant of the MUT increases, the resultant systematic error also increases, and the systematic error also increases with an increase in the frequency measurement error. Except for a few liquids, such as distilled water and glycerol, which exhibit a dielectric constant greater than 40, most common materials have a relatively low dielectric constant. Taking $\varepsilon_{r}=40$ as the boundary, when the frequency measurement error dΔf<1000 kHz, the systematic error of the measurement value is less than 0.625%, indicating that the scalar measurement method based on the short-ended transmission line has high measurement accuracy. The sensitivity of the sensor is defined as:

(11) $$S=\frac{1}{f_{0}}\frac{d\Delta f}{d\varepsilon_{r}}\times 100\%$$

where $f_{0}$ denotes the central operating frequency of the sensor. The sensitivity variation curve of the sensor is depicted in Figure 4, from which it can be seen that the sensitivity decreases with the increasing dielectric constant of the MUT. When $\varepsilon_{r}=1$, the sensitivity is still greater than 1%, demonstrating that the system features a high level of sensitivity.

Compared with other dielectric constant measurement methods using open-ended or short-ended transmission lines, the key distinction of the proposed method is that variations in dielectric constant are directly reflected in the reflection coefficient, and this relationship can be directly derived via an analytical expression. In contrast, conventional methods typically rely on the indirect fitting of the relationship between dielectric constant and reflection coefficient. In our preliminary work, we attempted to combine open-ended and short-ended transmission lines with parallel resistors to measure the dielectric constant of the MUT. However, this study found that open-ended transmission lines suffer from fringing field effects, which cause the effective length of the transmission line to extend outward. Furthermore, this effective length is not fixed and cannot be fully compensated for when open-ended and short-ended transmission lines are used in combination, resulting in deviations in the measurement results. Given that short-ended transmission lines are free of such fringing field effects, this work employs only short-ended transmission lines for measurements and has thus developed a complete system accordingly.

## 3. Non-Ideal Analysis

**Figure 5:**
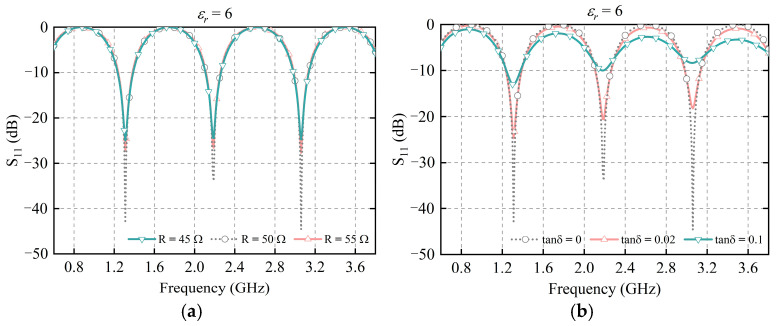
The circuit simulation results of the reflection coefficient under non-ideal conditions. (**a**) The impact of varying parallel resistance values; (**b**) the effect of the material intrinsic loss.

Although the feasibility of the scalar measurement method proposed in this paper has been verified through theoretical analysis in Section 2, the implementation conditions are relatively demanding, which necessitates the consideration of non-ideal conditions during experimental validation. Specifically, for a parallel resistance 50 Ω at the input end of the short-ended transmission line, ordinary chip resistors will exhibit significant deviations in actual resistance due to parasitic effects in the working frequency band of GHz. Even high-frequency resistors can experience resistance variation on the order of ±5%. Therefore, it is essential to analyze the impact of resistance variation on the input reflection coefficient. As shown in Figure 5a, by fixing $\varepsilon_{r}=6$ and varying the parallel resistance values to 45 Ω and 55 Ω, we can observe the amplitude variation in the reflection coefficient. The simulation results indicate that change in the parallel resistance only affects the resonance depth at the resonance frequency without causing any shift in the resonance frequency. This demonstrates that even if the resistance of the parallel resistor changes during operation, it will not affect the measurement results. This conclusion can also be derived from theoretical analysis. Assuming the resistance of the parallel resistor in Figure 2a is $R_{1}$, with a conductance of $G_{1}$, the input admittance $Y_{\mathrm{in}3}$ and the input reflection coefficient $\Gamma_{\mathrm{in}3}$ can be expressed as:

(12) $$Y_{\mathrm{in}3}=Y_{\mathrm{in}1}+G_{1}$$

(13) $$\Gamma_{\mathrm{in}3}=\frac{Y_{0}-\left(Y_{\mathrm{in}1}+G_{1}\right)}{Y_{0}+\left(Y_{\mathrm{in}1}+G_{1}\right)}$$

Since both $Y_{0}$ and $G_{1}$ are pure real numbers, and $Y_{\mathrm{in}1}$ is a pure imaginary number, derivative analysis is required to find the minimum point of the $\left|\Gamma_{\mathrm{in}3}\right|$. The minimum points of $\left|\Gamma_{\mathrm{in}3}\right|$ and ${\left|\Gamma_{\mathrm{in}3}\right|}^{2}$ are equivalent. For the sake of simplicity, $D\left(f\right)={\left|\Gamma_{\mathrm{in}3}\right|}^{2}$, differentiation of the squared magnitude of the reflection coefficient yields:

(14) $$D^{\prime }\left(f\right)=\frac{8Y_{0}^{2}G_{1}b_{s}}{{\left[{\left(Y_{0}+G_{1}\right)}^{2}+Y_{0}^{2}b_{s}^{2}\right]}^{2}}$$

In (14), the denominator is always greater than 0, so the only critical point for $D^{\prime }\left(f\right)=0$ is $b_{s}=0$. When $b_{s}<0$, $D^{\prime }\left(f\right)<0$, when $b_{s}>0$, $D^{\prime }\left(f\right)>0$, which indicates that $b_{s}=0$ is the point of local minimum for $\left|\Gamma_{\mathrm{in}3}\right|$. This demonstrates that the resonant frequency of the reflection coefficient remains unchanged with variations in the parallel resistance value, whereas the resonant depth varies accordingly. When $G_{1}=Y_{0}$, the local minimum point becomes the absolute minimum point.

During the ideal simulation of the circuit model, the influence of material loss on the reflection coefficient was neglected. In reality, all physical materials exhibit losses, and it is therefore essential to analyze the impact of material loss on the resonant frequency of the reflection coefficient. Setting the parallel resistance to R=50 Ω and the dielectric constant of the coaxial medium layer to $\varepsilon_{r}=6$, we simulated the variation in the input reflection coefficient for tanδ=0.02 and tanδ=0.1, and the simulation results are shown in Figure 5b. It can be observed that variations in material loss affect the resonance depth but do not shift the resonant frequency, which demonstrates that for the system based on resonance null positions discussed in this paper, variations in material loss do not affect the measurement results.

It is worth noting, however, that if one attempts to extract the loss tangent from the resonance depth, the resistive loading imposes a fundamental limitation on the detectable loss. In the equivalent circuit, the shunt resistor contributes a conductance $G_{R}=1/R$, while the MUT contributes a loss conductance $G_{MUT}=\omega \varepsilon_{r}\tan\delta$. When $G_{MUT}\leq G_{R}$,

(15) $$\tan\delta \leq \frac{1}{R\omega \varepsilon_{r}}$$

the reflection coefficient at resonance is dominated by the resistive loading rather than by the material absorption, and the measurement becomes insensitive to the intrinsic loss of the MUT. For the present system with R=50 Ω and an operating frequency around 2 GHz, this threshold is approximately $1.8\times 10^{-3}$ for air and decreases to about $2.3\times 10^{-5}$ for a material with $\varepsilon_{r}=80$. This trade-off is intrinsic to the scalar measurement strategy. The resistor that enables the formation of detectable resonant nulls also masks low-level loss information. For this primary purpose, the insensitivity to material loss is in fact advantageous, as it ensures that the resonance period, and consequently the extracted permittivity, remains robust against variations in material dissipation.

**Figure 6:**
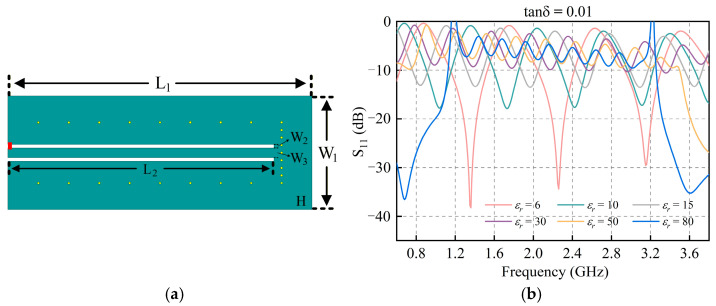
(**a**) The short-ended transmission line model based on a metallized coplanar waveguide; (**b**) the three-dimensional electromagnetic simulation results of the scattering parameters under different dielectric constant.

Traditional transmission lines such as microstrip lines and strip lines, due to the dielectric substrates used for structural fixation on one or both sides of the conductor, cannot meet the application scenarios where the transmission line is completely surrounded by dielectric material. If a coaxial transmission line is selected as the sensor probe, on the one hand, it is inconvenient to load the MUT between the inner and outer conductors of the coaxial line, on the other hand, custom-sized coaxial lines are not easy to connect with other devices. To address the practical issue that traditional transmission lines are difficult to directly adapt to the measurement method proposed in this paper, we introduce a novel transmission line based on an air coplanar waveguide. Compared with traditional coplanar waveguides, the air coplanar waveguide effectively eliminates the dielectric layer by increasing the thickness of the metal layer, thereby ensuring structural stability. In actual fabrication, the dielectric layer is not completely removed; instead, the surface of the dielectric layer is fully metallized to achieve an equivalent metallic structure. The model structure of the air coplanar waveguide is shown in Figure 6a, with the dielectric layer made of FR-4, a thickness of 1.60 mm, a dielectric constant of 4.4, and a loss tangent of 0.02. Since its surface is fully metallized, the electromagnetic parameters of the dielectric material do not affect the measurement principle of the short-ended probe. However, to meet the characteristic impedance of the transmission line in a vacuum environment as 50 Ω, these electromagnetic parameters will directly influence the dimensions of the air-based coplanar waveguide, with detailed dimensions shown in Table 1. During the metallization process, aside from the four edges of the dielectric material, all other surfaces are set as ideal electric boundaries, with the yellow circular area representing the metallized via, which enhances the electrical continuity of the upper and lower metallic surfaces.

Using HFSS 19.0 software, a three-dimensional electromagnetic simulation of the proposed short-ended transmission line model was conducted. During the simulation, a patch resistor with a resistance of 50 Ω was placed at the input end of the transmission line, with its length and width denoted as 2.54 mm and 1.27 mm, respectively, as illustrated in the red region of Figure 6a. Given the finite size of the patch resistor, the actual effective length of the short-ended transmission line is not exactly equal to 70.00 mm. Therefore, an approximation was made when calculating the dielectric constant, adjusting the value of l to be the preset length minus the width of the resistor, yielding l=68.73 mm. The simulation was carried out in environments where the coplanar waveguide was completely surrounded by $\varepsilon_{r}=6$, $\varepsilon_{r}=10$, $\varepsilon_{r}=15$, $\varepsilon_{r}=30$, $\varepsilon_{r}=50$ and $\varepsilon_{r}=80$, with the loss tangent set to tanδ=0.01. As shown in Figure 6b, the simulation results clearly indicate that as the dielectric constant increases, the resonant period of the reflection coefficient decreases, which is in accordance with theoretical expectations. Table 2 summarizes the first four resonant frequencies of the reflection coefficient for six different dielectric constants, as well as the average resonant frequency difference. Based on (9), the calculated values of the dielectric constant and the relative error were derived. The results indicate that for the six dielectric constants, the calculated values are very close to the preset values, with relative errors less than 1% for cases where the resonance period remains uniform. It is worth noting, however, that for extremely high dielectric constant values such as 50 and 80, the simulated reflection coefficient curves exhibit noticeable amplitude fluctuations and aperiodic distortions over the entire frequency band. This phenomenon primarily arises from the substantial deviation of the actual characteristic impedance of the coplanar waveguide from the 50 Ω port reference impedance under high-permittivity loading, as well as the weak excitation of higher-order modes. Nevertheless, the resonance nulls determined by the dominant mode in the lower-frequency range remain identifiable. By extracting the frequency spacing Δf between adjacent nulls within this locally stable region and substituting it into (9), reasonably approximate permittivity values can still be obtained, as listed in Table 2. This observation indicates that the proposed measurement principle retains local validity within a limited low-frequency bandwidth even under extreme permittivity conditions. For rigorous application of the theoretical model, however, the recommended effective measurement range is $\varepsilon_{r}\leq 30$, where the resonance period remains uniform and free from significant modal interference. Within this range, the short-ended transmission line-based dielectric constant sensor proposed in this paper exhibits high measurement accuracy.

## 4. System Design and Experimental Validation

**Figure 7:**
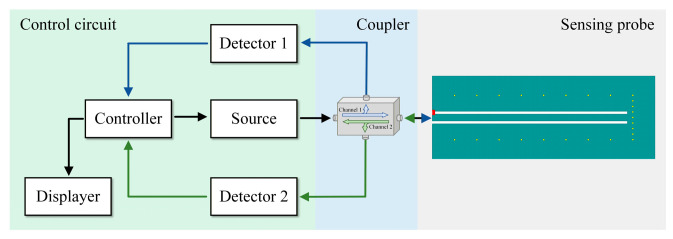
The block diagram of the working principle of the system.

Typically, a sensing probe requires a VNA for scattering parameters measurement. However, the measurement method proposed in this paper essentially involves scalar measurement of a single-port network, enabling the independent design of the entire sensor system. As shown in Figure 7, the block diagram of the working principle of the system comprises three main components: the control circuit, the broadband coupler, and the sensing probe. The control circuit is primarily responsible for signal generation, as well as the reception and processing of reflected signals. The broadband coupler separates the incident and reflected signals, whereas the sensing probe reflects electromagnetic signals carrying the dielectric constant information of materials through electromagnetic coupling with the surrounding medium. The entire system can independently measure the dielectric constant of materials without reliance on any assistive devices.

**Figure 8:**
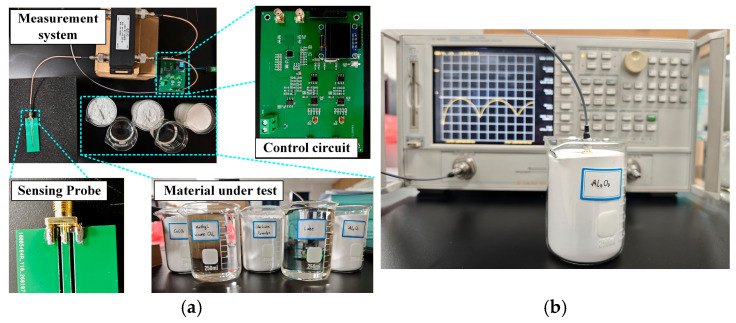
The experimental testing process. (**a**) Prototype of the sensor system; (**b**) validation of the sensor probe effectiveness using a VNA.

As shown in Figure 8a, the physical prototype of the system and the measurement process are presented. The control circuit is fabricated using PCB technology (Migdal Haemek, Israel), with the control chip and signal source chip being STM32 and ADF4351, respectively. The ADF4351 wideband synthesizer is controlled by the STM32 microcontroller via a three-wire serial interface. Together, they can generate a program-controlled frequency sweeping signal within the frequency band of 35 MHz to 4.4 GHz, with a minimum step size of 100 kHz and the fastest sweeping speed of 1 ms per point. To evaluate the frequency stability of the swept source, the output frequency at a fixed setting of 2.0 GHz was monitored over a period of 5 min using a spectrum analyzer. The measured frequency drift was within ±150 kHz, which corresponds to a relative frequency error of less than 0.0075 percent. This level of stability is sufficient for the proposed measurement method, as the frequency measurement error is dominated by the step size of 1 MHz rather than the long-term drift. The broadband coupler separates the incident and reflected signals, directing them to Detector 1 and Detector 2, both of which are AD8362 logarithmic power detectors. The AD8362 provides a DC output voltage proportional to the logarithm of the input power, with a dynamic range of approximately 60 dB from −52 dBm to +8 dBm. This setup allows for the direct conversion of RF signals to baseband voltage. The baseband voltage signal is fed into the control chip for AD conversion and corresponding signal processing, with the result displayed on the monitor. Considering the operating characteristics of the broadband coupler, the actual working frequency band of the control circuit is 0.6 GHz to 3.8 GHz, with a frequency step of 1 MHz, and the duration of each frequency sweep point is 30 ms, during which sampling occurs at the midpoint of each sweep point’s duration. The sensing probe utilizes an FR-4 dielectric substrate, which is metallized on its surface, with the substrate material and parameters identical to those used in the simulation.

The test materials used in this study include two liquid materials and three chemical powder reagents, all of which are insulating materials with stable dielectric constant at high frequencies. The liquid test materials are transformer oil (10#) and methyl silicone oil (500C.S), while the solid test materials consist of aluminum oxide powder (Al_2_O_3_), calcium carbonate powder (CaCO_3_), and medical-grade talcum powder. It is noteworthy that air, which completely envelops the sensing probe, can also be considered a test material.

**Figure 9:**
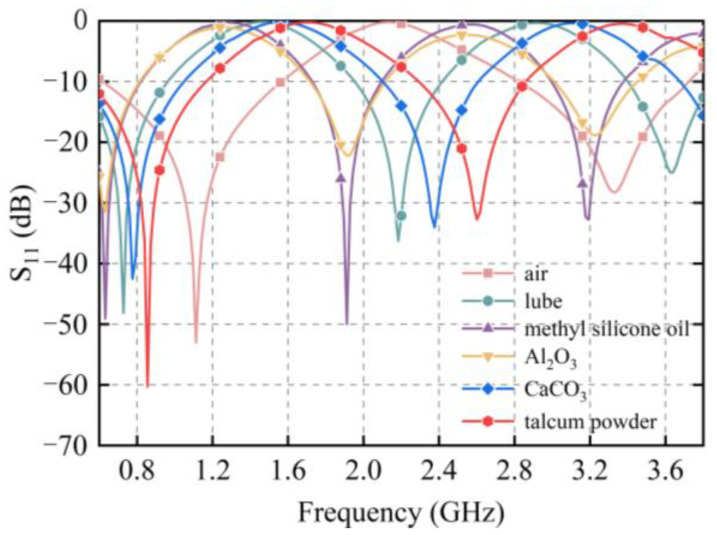
The scattering parameter measurement results of six different samples using the VNA.

During the experiment, the sensing probe is fully immersed in the liquid or completely inserted into the solid powder being tested. All measurements are carried out at room temperature (25 °C). After each measurement, the sensing probe was rinsed thoroughly with distilled water and dried in a laboratory oven at 60 °C for 20 min to remove any residual moisture before the next measurement. To validate the effectiveness of the proposed system, a VNA was used for auxiliary measurement, with the VNA model was Agilent (Santa Clara, CA, USA) 8720ET, as shown in Figure 8b. The measurement result from the VNA is depicted in Figure 9, where it can be observed that the reflection coefficient exhibits different resonance periods when the sensing probe is inserted into different liquid or solid materials. The dielectric constant of the test material can be calculated from these resonance periods. The sensor system cannot directly display the curve change in the scattering parameters. Therefore, the resonant frequency points measured by the VNA and the sensor system are listed in Table 3. Reference values are taken from the dielectric constant data reported in Von Hippel and subsequent microwave dielectric constant measurement studies [20,21]. The resonant frequency of each material is obtained from three independent measurements. The results are presented as the mean value accompanied by the standard deviation (Std. Dev) and maximum deviation (Max. Dev) to quantitatively demonstrate the measurement repeatability. It can be observed that the measurement results from the VNA are very close to those from the sensor system, indicating the effectiveness and feasibility of the designed independent system. For air, which has the most stable dielectric constant, the measurement error of both the VNA and the system are less than 3%, proving that the dielectric constant measurement method based on a short-ended transmission line proposed in this paper has high measurement accuracy. The measured value of the dielectric constant for transformer oil and methyl silicone oil falls between the given reference values, as liquid materials can completely isolate the air surrounding the sensing probe. For solid material, the value of the dielectric constant is related to the size of the solid particles and the packing density of the material. Among the three solid materials selected for the experiment, the Al_2_O_3_ has the smallest particle size, and its measured value falls within the reference range, which is considered reasonable. In contrast, the measured values for CaCO_3_ and talcum powder are both lower than the reference values, which can be attributed to the larger particle sizes of these two materials, making the influence of air gaps more pronounced and consequently lowering the measured values of dielectric constant. This research result indicates that the sensor system proposed in this paper is more suitable for powdered solid or liquid material with stable dielectric constant and insulation at high frequencies, and for powdered solid material, those with smaller particle sizes yield better measurement results.

## 5. Conclusions

This paper presents a novel resonance-period-based dielectric constant sensor system employing a short-ended transmission line. The theoretical derivation of the proposed method is elaborated, and an analysis of non-ideal effects as well as experimental validation are also conducted. The key innovation of this work lies in using the resonance period of the scattering parameters to reflect changes in the dielectric constant of the MUT. This approach enables the direct calculation of the dielectric constant via an analytical formula, eliminating the need for numerical fitting or calibration curves. In contrast, traditional microwave methods indirectly infer the dielectric constant by fitting measurement results based on changes in the frequency points or phase of the scattering parameters. A dedicated integrated control circuit was designed to meet the measurement requirements, forming a complete sensor system with a broadband coupler and a sensing probe. Experimental results demonstrate that the proposed system is suitable for powdered solids and liquid materials with stable dielectric constant at high frequencies, achieving high measurement accuracy for solid materials with small particle sizes. The system features advantages such as low cost, high precision, and portability, indicating broad application prospects in the field of radio frequency identification.

## Figures and Tables

**Table 1:** The detailed dimensions of the short-ended transmission line (mm).

Dimension	$L_{1}$	$L_{2}$	$W_{1}$	$W_{2}$	$W_{3}$	H
Value	80.00	70.00	30.00	1.00	2.45	1.60

**Table 2:** The resonance frequency points (GHz) and calculated values of dielectric constant under six different permittivities.

	$f_{1}$	$f_{2}$	$f_{3}$	$f_{4}$	Δf	$\varepsilon_{r\_cal}$	Error (%)
$\varepsilon_{r}=6$	1.36	2.26	3.15		0.895	5.95	0.83
$\varepsilon_{r}=10$	1.04	1.73	2.42	3.11	0.690	10.00	0.00
$\varepsilon_{r}=15$	0.84	1.40	1.97	2.53	0.562	15.08	0.53
$\varepsilon_{r}=30$	0.99	1.38	1.78	2.17	0.393	30.84	2.8
$\varepsilon_{r}=50$	1.07	1.37	1.68	1.98	0.293	55.48	10.96
$\varepsilon_{r}=80$	1.31	1.57	1.81	2.05	0.247	78.07	2.41

**Table 3:** Comparison of VNA auxiliary measurement results with the system measurements results.

		VNA Measurement Results				System Measurement Results			
Material	Reference Value	$\Delta \bar{f}(\mathrm{GHz})$	Std. Dev (GHz)	Max. Dev (GHz)	$\varepsilon_{r\_cal}$	$\Delta \bar{f}(\mathrm{GHz})$	Std. Dev (GHz)	Max. Dev (GHz)	$\varepsilon_{r\_cal}$
air	1	2.216	0.002	0.003	0.970	2.214	0.004	0.004	0.972
transformer oil	2.1–2.3	1.448	0.003	0.003	2.272	1.448	0.005	0.005	2.272
Al_2_O_3_	2.7–3.2	1.304	0.003	0.003	2.801	1.304	0.003	0.004	2.801
CaCO_3_	2.9–3.8	1.600	0.002	0.003	1.861	1.602	0.003	0.003	1.856
talcum powder	2.5–3.4	1.744	0.002	0.003	1.566	1.747	0.004	0.004	1.561
methyl silicone oil	2.7–3.0	1.280	0.003	0.003	2.907	1.280	0.005	0.005	2.907

## Data Availability

The data presented in this study are available on request from the corresponding author due to privacy/ethical/legal restrictions.
